# Prognostic Role of Hypoxia-Inducible Factor-2α Tumor Cell Expression in Cancer Patients: A Meta-Analysis

**DOI:** 10.3389/fonc.2018.00224

**Published:** 2018-06-11

**Authors:** Eloy Moreno Roig, Ala Yaromina, Ruud Houben, Arjan J. Groot, Ludwig Dubois, Marc Vooijs

**Affiliations:** ^1^Department of Radiotherapy (MAASTRO)/GROW – School for Developmental Biology and Oncology, Maastricht University, Maastricht, Netherlands; ^2^Department of Radiation Oncology, MAASTRO Clinic, Maastricht, Netherlands

**Keywords:** cancer, hypoxia-inducible factor-2, meta-analysis, prognosis, endothelial PAS domain protein-1

## Abstract

Hypoxia-inducible factor-2α (HIF-2α) plays an important role in tumor progression and metastasis. A number of studies have evaluated the correlation between HIF-2α overexpression and clinical outcome in cancer patients but yielded inconsistent results. To comprehensively and quantitatively summarize the evidence on the capability of HIF-2α to predict the prognosis of cancer patients with solid tumors, a meta-analysis was carried out. Renal cell carcinoma (CC-RCC) was separately analyzed due to an alternative mechanism of regulation. Systematic literature searches were performed in PubMed and Embase databases for relevant original articles until February 2018. Forty-nine studies with 6,052 patients were included in this study. The pooled hazard ratios (HRs) with corresponding confidence intervals were calculated to assess the prognostic value of HIF-2α protein expression in tumor cells. The meta-analysis revealed strong significant negative associations between HIF-2α expression and five endpoints: overall survival [HR = 1.69, 95% confidence interval (95% CI) 1.39–2.06], disease-free survival (HR = 1.87, 95% CI 1.2–2.92), disease-specific survival (HR = 1.57, 95% CI 1.06–2.34), metastasis-free survival (HR = 2.67, 95% CI 1.32–5.38), and progression-free survival (HR = 2.18, 95% CI 1.25–3.78). Subgroup analyses revealed similar associations in the majority of tumor sites. Overall, these data demonstrate a negative prognostic role of HIF-2α in patients suffering from different types of solid tumors.

## Introduction

Hypoxia is a common feature of most of solid tumors resulting from an imbalance between oxygen supply and consumption by tumor cells. Hypoxic tumor areas are characterized by a disrupted vasculature causing inefficient oxygen and nutrient supply to neighboring cells (1). Hypoxia is one of the key factors in inducing the development of resistant cells with an aggressive phenotype (2), which leads to poor prognosis in patients and decreases the efficacy of chemoradiotherapy (3, 4). Accurate measurement of tumor hypoxia in patients together with the design of novel anti-hypoxia treatments has largely been a major goal in cancer research (5–8).

Hypoxia triggers important cellular stress responses allowing tumor cells to survive under extreme conditions, including the stabilization of the hypoxia-inducible factor (HIF) proteins (9, 10). Under normoxic conditions, prolyl-hydroxylation promotes HIF-α degradation *via* the von Hippel–Lindau (VHL) ubiquitin/proteasome pathway. Under hypoxia, this regulation is suppressed, leading to the stabilization of three independent HIF-α subunits (HIF-1α, HIF-2α, and HIF-3α) that dimerize with the constitutively expressed HIF-1β and activate the transcription of genes *via* hypoxia responsive elements in their promoter region. The HIF-2α protein, also named endothelial PAS domain protein-1, is equally oxygen regulated as its counterpart HIF-1α, and both present over 50% similarity in their amino acid sequence identity (11). In physiologic conditions, HIF-1α has a broad activity in several tissues which contain hypoxic regions, while HIF-2α is more restricted to specific cell types, e.g., kidney, lung, and heart (12). HIF proteins distinctly contribute to the upregulation of genes involved in proliferation, glucose metabolism, and angiogenesis and genes involved in invasion and metastasis in different types of cancer (13). HIF isoforms also differ in their ability to promote treatment resistance in cancer by playing highly divergent or even opposite roles, leading to distinct clinicopathologic features and prognosis (14). Specific activity of HIF-2α differently contributes to total HIF target gene expression among many types of cancers, which may influence the characteristics of these tumors and the outcome of patients (15). To date, much effort has been made to better understand the roles of HIF-2α in cancer and the consequences for patients suffering from high-HIF-2α expressing tumors. So far, there is clear evidence suggesting that HIF-2α is a crucial protein for the development and progression of many types of cancer. Indeed, HIF-2α seems to be crucial in regulating multiple aspects in cancer, including cell proliferation, apoptosis, epithelial-to-mesenchymal transition, cell metabolism, angiogenesis, and resistance to therapy (16, 17). Hypoxia-induced HIF-2α expression and its subsequent chain of events make this protein a relevant marker of tumor hypoxia and a promising target for anticancer therapies with novel inhibitors.

Several clinical studies describe the prognostic value of HIF-1α in cancer, including the elaboration of many meta-analyses for several kinds of cancer and different HIF-1α polymorphisms (18–20). For some of the clinical studies included in these meta-analyses, prognostic data on HIF-2α can be found which often differed from HIF-1α data (21). In addition, other studies specifically looked at the role of HIF-2α independently of HIF-1α expression. Nonetheless, many discrepancies are seen among the investigations that were performed during the last years, in which HIF-2α has been reported to be either a positive or negative prognostic factor in cancer. The purpose of this meta-analysis was to provide an updated comprehensive analysis regarding the prognostic value of HIF-2α expression in solid tumors.

## Materials and Methods

### Literature Search

**Figure 1:**
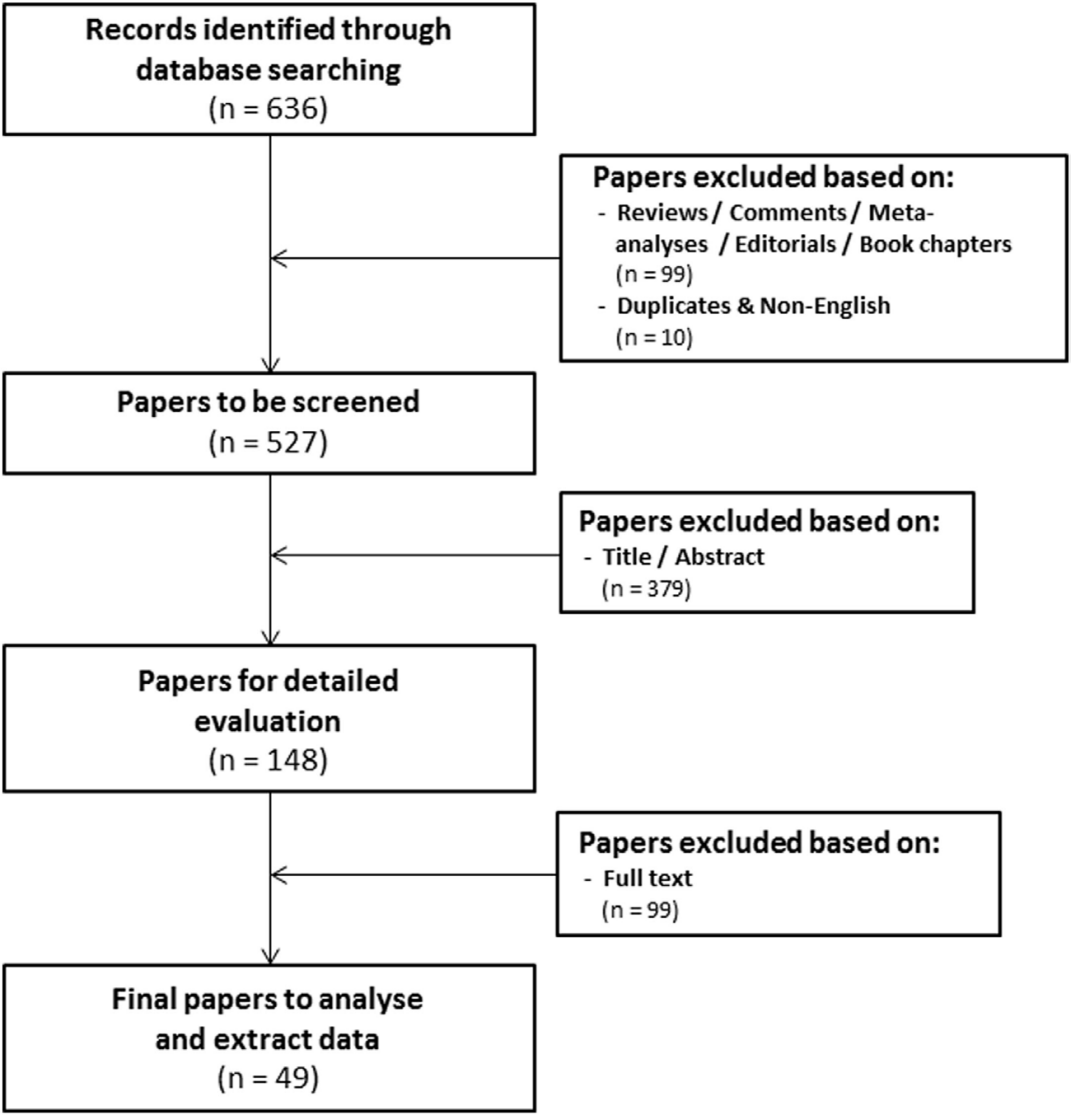
Flowchart of selecting articles describing the association between tumoral hypoxia-inducible factor-2α expression and prognosis.

The research question of this meta-analysis was defined as follows: “what is the prognostic value of tumoral HIF-2α expression in patients with solid tumors?” Meta-analysis included patients with solid tumors of different types (Table 1) independent of stage and/or grade or treatment modality. Various treatment outcomes (see below) were compared between patients with high and low tumoral HIF-2α expression. PubMed and Embase were used to identify studies that investigated the prognostic significance of HIF-2α in solid tumors to be included in this meta-analysis. The search for literature was performed including papers published until February 1, 2018. Three main key words were identified to address the research question of this meta-analysis, i.e., *prognosis, cancer*, and *HIF2*. Several combinations of the selected keywords (in any of the formulations or truncations) were tested as free text searches to identify potential articles to be included in this meta-analysis (Supplementary File S1 in Supplementary Material). A total of 636 papers were identified from both databases (Figure 1).

**Table 1:** Summary of results of subgroup meta-analyses of different organ sites.

Organ site	Overall survival	Disease-free survival	Disease-specific survival	Locoregional control	Metastasis-free survival	Progression-free survival
Bladder				**0.51 (0.33–0.77)**^a^		
Brain	**3.78 (1.64–8.75)**^a^					
Breast	1.18 (0.95–1.47)		**2.3 (1.3–4.1)**^a^	**1.6 (1–2.4)**^a^		
Cartilage	**4.13 (1.47–11.58)**^a^					
Cervix				**1.53 (1.14–2.03)**^a^		
Colorectal	1.46 (0.7–3.04)	1.86 (0.89–3.92)^a^	0.8 (0.61–1.05)^a^	0.54 (0.25–1.17)^a^		
Endometrium	**5.72 (1.51–21.6)**^a^					
Head and neck	**1.55 (1.24–1.92)**	1.41 (0.99–2)	1.45 (0.8–2.64)	**1.94 (1.43–2.63)**		
Kidney	0.61 (0.27–1.35)		1.21 (0.57–2.6)	0.54 (0.11–2.57)	**0.08 (0.03–0.27)**^a^	0.75 (0.32–1.73)
Liver	1.06 (0.43–2.61)	1.52 (0.82–2.84)^a^		1.22 (0.87–1.83)^a^		
Lung	**2.15 (1.65–2.81)**	**8.47 (3.26–22.06)**^a^				
Ovarium	**2.72 (1.27–5.83)**^a^					
Pancreas	**2.11 (1.38–3.24)**					**1.67 (1.26–2.21)**^a^
Prostate					**2.44 (1.07–5.57)**^a^	**2.94 (1.99–4.36)**^a^
Salivary glands	**4.52 (1.06–19.3)**^a^			3.64 (0.72–18.42)^a^	3.36 (0.89–12.66)^a^	
Skin	**3.21 (1.15–8.97)**^a^		**3.81 (1.66–8.78)**^a^			
Soft tissues			**1.53 (1.03–2.28)**^a^			
Stomach	**1.7 (1.25–2.32)**		**1.6 (1–2.4)**^a^			

*The hazard ratio (HR) with 95% confidence interval is shown. Highlighted numbers indicate statistically significant association between hypoxia-inducible factor-2α expression and prognosis (*p* < 0.05)*.

*^a^When HR was available from only one paper, the values were adopted from that single paper (21–69)*.

### Screening of Papers

Three independent reviewers assessed the eligibility of the studies. The first round of screening was based on the title and abstract (Eloy Moreno Roig and Arjan J. Groot). 99 review articles without original data, conference records, commentaries, meta-analyses, editorials, or book chapters were excluded, as were 10 overlapping or non-English papers. The total number of papers for further screening was thereby reduced to 527 (Figure 1). The second round consisted of a detailed evaluation of the full-text (Eloy Moreno Roig and Ala Yaromina).

In order to be included in the meta-analysis, a study had to fulfill predetermined inclusion/exclusion criteria: (1) only solid primary tumors of various types were included, (2) all endpoints were included with the minimal median follow-up of 1 year, (3) all treatments were included, (4) none of the patient populations were excluded by distinctive diagnosis, tumor grade, or tumor stage, and (5) pre-treatment protein expression by immunohistochemistry was the only technique for HIF-2α detection included. Discrepancies between the included papers by both reviewers were discussed and consensus was reached on all. A total of 49 papers were included in the meta-analysis (Figure 1) (21–69).

### Data Extraction

Reported parameters were extracted from each paper, i.e., the number and origin of patients, treatment modalities, tumor organ, tumor stage, tumor type, group dichotomization, antibody supplier, expression pattern, cellular localization, positivity in macrophages, and outcome variables (see below). The univariate hazard ratio (HR) and 95% confidence interval (95% CI) were directly obtained from the information available in the text. If not reported, the method from Tierney et al. was used to calculate HR and thereby assess prognostic value of HIF-2α expression (70). Multivariate HR was only considered when the univariate HR could not be estimated. Authors were contacted to obtain additional data when not all the information was reported for estimating HR.

Kidney cancer was excluded from the main analysis because the loss of the VHL gene, a common mutation in CC-RCC, results in the stabilization of HIFs independent of oxygen, i.e., an alternative mechanism of HIF-2α activation in cancer absent in the majority of other solid tumors (71). Therefore, prognostic value of HIF-2α in this tumor type has been analyzed separately.

### Quality Assessment

The methodological quality of the included papers was evaluated with an adjusted version of the Newcastle–Ottawa scale (NOS) to better suit the study design of the included papers (Supplementary File S2 in Supplementary Material) (72). The NOS was proposed in the 2011 version of the Cochrane Collaboration handbook being an easy method to evaluate the methodological quality of cohort studies (available at http://www.ohri.ca/programs/clinical_epidemiology/oxford.asp).

### Statistical Analysis and Sensitivity Analysis

Distribution and frequencies of the extracted data parameters were analyzed using SPSS (version 22). Meta-analysis was performed using R statistical software with the Metafor Library (version 2.0-0) (73). Fixed-effect modeling was performed when no statistical significant heterogeneity between studies was observed. When the heterogeneity between studies was statistically significant (*p* < 0.05), random-effects modeling was applied based on the DerSimonian and Laird method (74). The inverse variance of each study was used to assign an independent weight value. Six different endpoints were considered for the analysis: overall survival (OS), disease-free survival (DFS), locoregional control (LC), disease-specific survival (DSS), metastasis-free survival (MFS), and progression-free survival (PFS). Sensitivity analysis was performed by analyzing subgroups of studies based on organ site. We assessed the possibility of publication bias and heterogeneity among studies by generating and visually analyzing funnel plots. Asymmetric funnel plots and studies outside the pyramid suggest heterogeneity between them.

## Results

This meta-analysis includes a total number of 6,052 patients across 49 independent studies (21–69). The median follow-up time ranged between 27 and 391 months and mostly included only a small number of patients (median 90, range 21–695). Selected papers were published between 2001 and 2017 of which 56% were published after 2010. Depending on the study, patients followed different treatment schedules. In most of the selected studies, patients were treated with surgery alone (56%), in combination with either chemotherapy (8%) or standard radiotherapy (10%), or a combination of all three modalities (14%). Other treatment alternatives such as hormonal therapy and tyrosine-kinase inhibitors were used in 8% of the studies. Overall, the majority of the patients (61%) were treated with surgery alone. Most studies report on head and neck and kidney cancer patients (both 18%) followed by colon, liver, pancreas, and lung cancer patients (all of them 8%). By contrast, cancers of the bladder, cartilage, cervix, endometrium, ovarian, and salivary glands were only described once.

Immunohistochemical staining of HIF-2α was most commonly performed using the EP190b Ab (38%) from Novus Biologicals. Other studies used anti-HIF2α antibodies obtained from other suppliers. Cytoplasmic expression of HIF-2α was described in 14% of the studies and 18% were positive in the nucleus. A combination of both positive cytoplasmic and nuclear staining was reported in 46% of the studies. HIF-2α expression was quantified using different methods and patient stratification into groups with low and high tumoral HIF-2α expression was performed using different thresholds. Taken together, 45% of the total tumors were classified as expressing high levels of HIF-2α. Also, 26% of these studies stated positive staining in macrophages.

**Figure 2:**
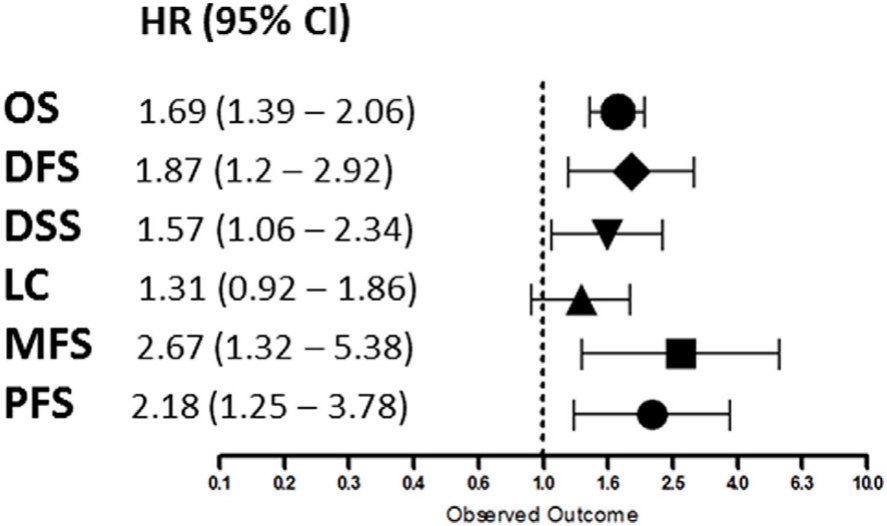
Summary of the overall hazard ratios (HRs) for different endpoints. Symbols represent the HR and horizontal bars the 95% confidence interval (95%CI) (21–69).

Overall, patients suffering from tumors with high HIF-2α expression had a worse treatment outcome (Figure 2). This association was significant for OS (*p* < 0.0001), DFS (*p* = 0.0057), DSS (*p* = 0.0249), MFS (*p* = 0.0061), and PFS (*p* = 0.0058). No association was found between HIF-2α expression and LC (*p* = 0.1281). Subgroup analyses based on tumor type and treatment option were not performed due to the low number of studies. Studies on renal cell cancer were eliminated from the overall HR estimation as HIF-2α plays a different role in this cancer type (71). Funnel plots demonstrated systematic heterogeneity for almost all the endpoints. This can be due to publication bias, variation across the reports, or small number of studies (Figure S1 in Supplementary Material).

### Overall Survival

**Figure 3:**
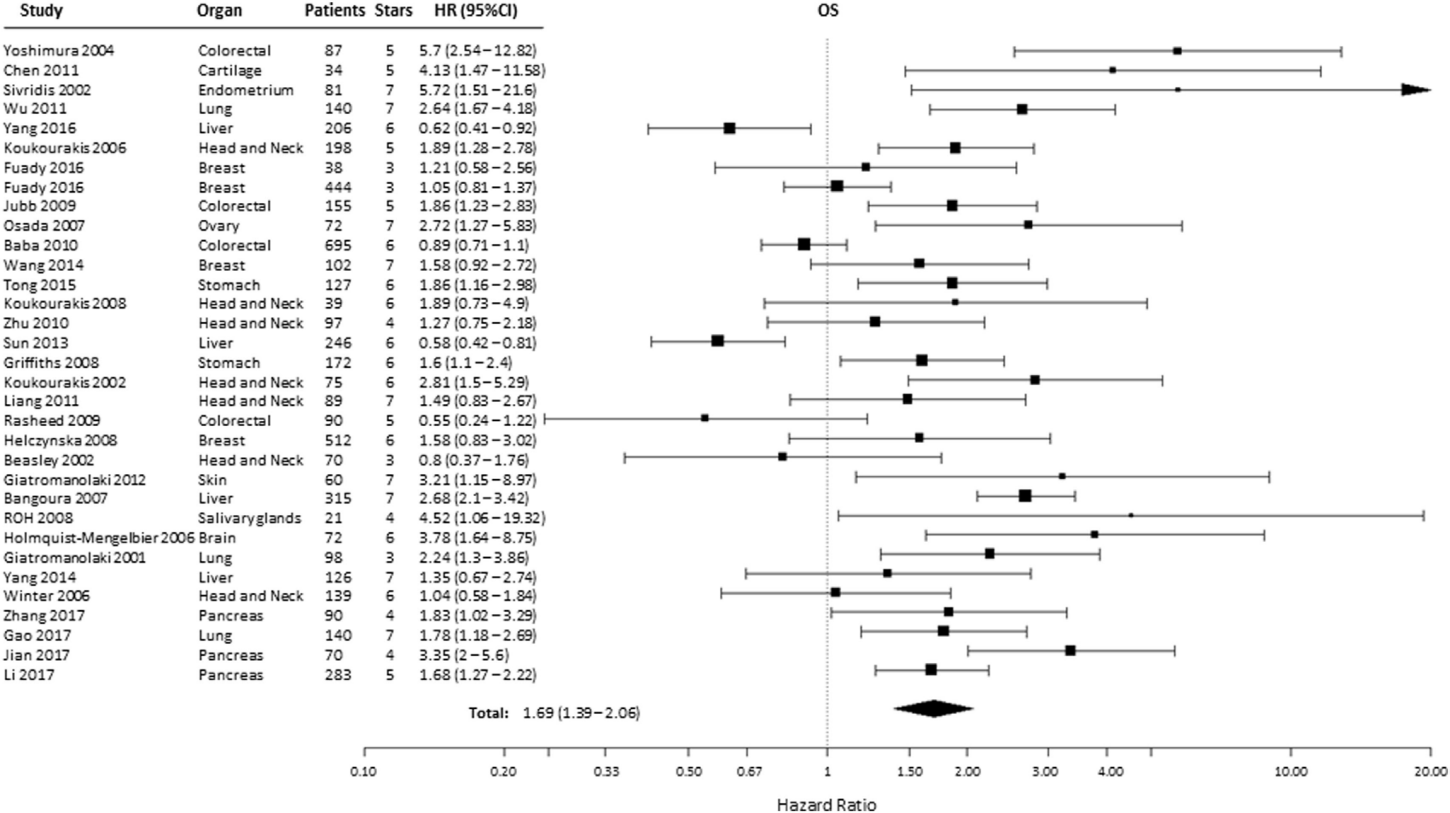
Forest plot of hazard ratios (HRs) with 95% confidence interval (95% CI) (horizontal bars) for the association of hypoxia-inducible factor-2α expression and overall survival (OS). Symbol size represents the assigned weight of the study (21, 24, 26, 28, 30, 31, 33–35, 37, 38, 41–43, 45, 46, 48, 49, 52, 54–57, 59, 60, 62–64, 66–69).

A total of 38 from the selected 49 studies investigated the association between HIF-2α and OS. All the necessary information to estimate the HR could not be obtained from two papers and were therefore not included in the analysis (Table S1 in Supplementary Material) (21, 23, 24, 26–31, 33–35, 37–39, 41–43, 45, 46, 48, 49, 51, 52, 54–57, 59, 60, 62–69). Based on these studies, high HIF-2α expression was statistically significantly associated with a decreased OS (HR = 1.69, 95% CI 1.39–2.06, *p* < 0.0001, Figure 3). Subgroup analysis based on the different organ sites indicated a similar negative association between tumoral HIF-2α expression and OS: head and neck (HR = 1.55, 95% CI 1.24–1.92, *p* < 0.0001), lung (HR = 2.15, 95% CI 1.65–2.81, *p* < 0.0001), stomach (HR = 1.71, 95% CI 1.25–2.32, *p* = 0.0007), and pancreas (HR = 2.11, 95% CI 1.38–3.24, *p* = 0.0006). By contrast, no association between tumoral HIF-2α expression and OS was observed for breast (HR = 1.18, 95% CI 0.95–1.47, *p* = 0.1225), colon (HR = 1.46, 95% CI 0.7–3.07, *p* = 3121), liver (HR = 1.06, 95% CI 0.43–2.61, *p* = 0.89), and kidney (HR = 0.61, 95% CI 0.27–1.35, *p* = 0.2239) cancer (Table 1).

### Disease-Free Survival

**Figure 4:**
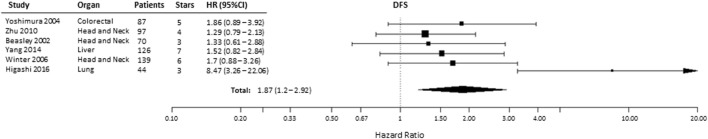
Forest plot of hazard ratios (HRs) with 95% confidence interval (95% CI) (horizontal bars) for the association between hypoxia-inducible factor-2α expression and disease-free survival (DFS). Symbol size represents the assigned weight of the study (36, 37, 54, 66, 67, 69).

Effect of pre-treatment expression of HIF-2α on DFS could be evaluated in six studies (36, 37, 54, 66, 67, 69). Overall, high HIF-2α expression was significantly associated with a decreased DFS (HR = 1.87, 95% CI 1.2–2.92, *p* = 0.0057, Figure 4). Subgroup analysis indicated that elevated HIF-2α levels were marginally significantly associated with DFS in head and neck cancer (HR = 1.41, 95% CI 0.99–2, *p* = 0.0577) (Table 1).

### Disease-Specific Survival

**Figure 5:**
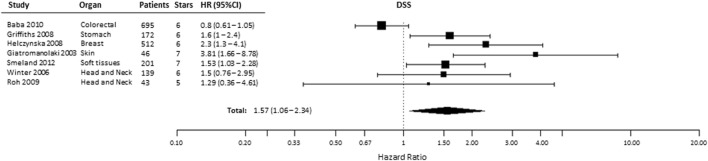
Forest plot of hazard ratios (HRs) with 95% confidence interval (95% CI) (horizontal bars) for the association between hypoxia-inducible factor-2α expression and disease-specific survival (DSS). Symbol size represents the assigned weight of the study (24, 32, 34, 35, 58, 61, 66).

A total of 12 studies evaluated the association of HIF-2α expression with DSS, of which 2 studies provided incomplete data to estimate the HR (Table S1 in Supplementary Material) and 3 were excluded for being CC-RCC. In the remaining seven studies, patients suffering from tumors with high HIF-2α had significantly shorter DSS (HR = 1.57, 95% CI 1.06–2.34, *p* = 0.0249, Figure 5) (24, 25, 32, 34, 35, 47, 49, 50, 53, 58, 61, 66). Subgroup analysis by organ site revealed not association between high HIF-2α expression and worse DSS in tumors of the head and neck (HR = 1.45, 95% CI 0.8–2.64, *p* = 0.2219) and kidney (HR = 1.21, 95% CI 0.57–2.6, *p* = 0.6138) cancer (Table 1).

### Locoregional Control

**Figure 6:**
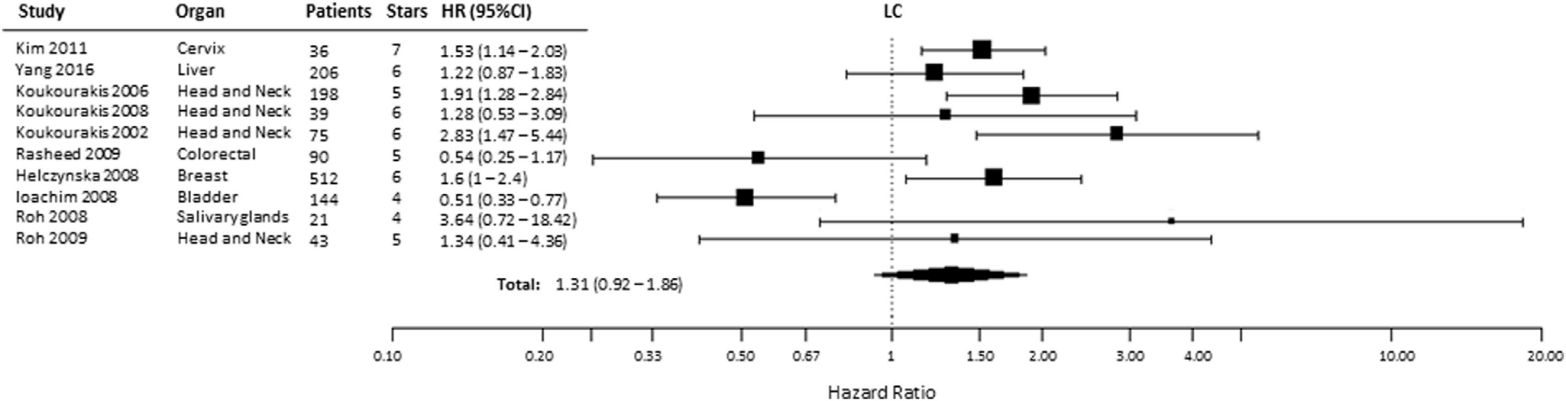
Forest plot of hazard ratios (HRs) with 95% confidence interval (95% CI) (horizontal bars) for the association between hypoxia-inducible factor-2α expression and locoregional control (LC). Symbol size represents the assigned weight of the study (35, 40, 44–46, 52, 56–59).

Ten studies were included to analyze the association of HIF-2α expression with risk of LC. High tumoral HIF-2α expression was not associated with a higher risk of locoregional recurrences compared with patients with low expression of HIF-2α in tumors (HR = 1.31, 95% CI 0.92–1.86, *p* = 0.1281, Figure 6) (35, 40, 44–46, 52, 56–59). However, subgroup analysis for the association between high HIF-2α expression in tumors and worse LC was significant in head and neck tumors (HR = 1.94, 95% CI 1.43–2.63, *p* < 0.0001). No significant association was observed in kidney tumors (HR = 0.54, 95% CI 0.11–2.57, *p* = 0.4409) (Table 1).

### Metastasis-Free Survival

**Figure 7:**
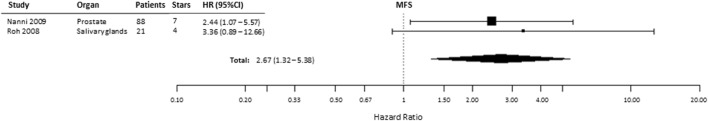
Forest plot of hazard ratios (HRs) with 95% confidence interval (95% CI) (horizontal bars) for the association between hypoxia-inducible factor-2α expression and metastasis-free survival (MFS). Symbol size represents the assigned weight of the study (53, 57).

Based on the available data reported in two studies, we analyzed the relationship of HIF-2α expression with MFS (53, 57). We found that the pooled HR for MFS was 2.67 (95% CI 1.32–5.38, *p* = 0.0061), indicating that HIF2α expression is a negative prognostic factor for MFS in patients with prostate and salivary gland cancer (Figure 7). In kidney cancer, one paper reported an inverse correlation between HIF-2α positivity and MFS (HR = 0.08, 95% CI 0.03–0.27, *p* < 0.001) (23).

### Progression-Free Survival

**Figure 8:**
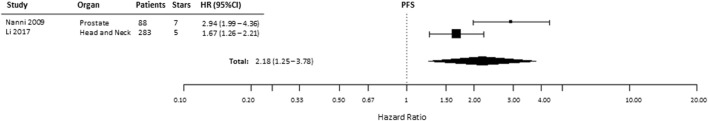
Forest plot of hazard ratios (HRs) with 95% confidence interval (95% CI) (horizontal bars) for the association between hypoxia-inducible factor-2α expression and progression-free survival (PFS). Symbol size represents the assigned weight of the study (48, 53).

Progression-free survival was reported in 5 of 49 included studies, of which 1 study provided incomplete data (Table S1 in Supplementary Material) and 2 described patients with renal cell cancer (29, 39, 48, 51, 53). Similar to the other endpoints, PFS was significantly shorter (HR = 2.18, 95% CI 1.25–3.78, *p* = 0.0058) in patients with tumors expressing high levels of HIF-2α (Figure 8). In kidney cancers, these data showed no association between HIF-2α expression and PFS (HR = 0.75, 95% CI 0.32–1.72, *p* = 0.498) (Table 1).

### High-Quality Papers

This meta-analysis used an adjusted version of the NOS to evaluate the quality of a study. The scores of this quality assessment ranged between 1 and 7 stars, i.e., the maximum, awarded per study. Approximately 78% of these studies were considered as high-quality studies, i.e., with a number of stars greater or equal to 5.

## Discussion

There is growing evidence that overexpression of HIF-2α in cancer can contribute to differences in treatment outcome between patients. Although HIF-2α oncogenic activity has been proven, there is significant variability in its value as a biomarker for patient’s prognosis. In view of its role in regulating oncogenic processes triggered by hypoxia, the evaluation of its prognostic value in cancer is of great clinical importance, which may lead to a more accurate patient prognosis and the generation of targeted therapies in the future. This meta-analysis is the first complete overview to summarize all reported clinical studies investigating the impact of HIF-2α expression on treatment outcome in solid tumors.

Here, we show that high HIF-2α levels in cancer are correlated with worse prognosis for OS, DFS, DSS, MFS, and PFS. No association between HIF-2α expression and LC was found. These data suggest that HIF-2α might not be involved in treatment resistance directly but is indicative of more malignant phenotype with greater metastatic potential. Subgroup analyses were performed to explore the source of heterogeneity based on different organ sites. We found that this variable did not alter the prognostic value of HIF-2α for most of the endpoints assessed.

We excluded CC-RCCs from the main analysis due to a different regulatory mechanism of HIF-2α, which might affect the final outcome. A common mutation in CC-RCC is the loss of the VHL gene, which results in the stabilization of HIFs upon normoxia. This oncogenic process is specific for CC-RCC with an abundance of 80% in patients, resulting in an alternative mechanism of HIF-2α activation in cancer (71). However, the negative prognostic value of HIF-2α does not change when CC-RCC is included in the overall meta-analysis (data not shown). In CC-RCC, we found that HIF-2α is a positive prognostic biomarker for MFS only (Table 1). Subcellular localization might also affect the prognostic significance of HIF-2α in CC-RCC as previously noted (75). Their data show that high cytoplasmic expression of HIF-2α was significantly associated with poor DSS which is consistent with our findings. By contrast, high nuclear expression of HIF-2α is associated with better DSS in patients (Table S2 in Supplementary Material). Therefore, it might be that subcellular localization of HIF-2α is crucial in determining the prognostic value in CC-RCC patients, which requires further investigation. Another study demonstrated that HIF-2α can be used as a predictive biomarker related to drug selection for CC-RCC patients treated with sunitinib and sorafenib (51). Therefore, assessment of subgroup categories is necessary to better evaluate the prognostic and predictive value of HIF-2α in CC-RCC.

Previous studies have shown that increased infiltration of tumor-associated macrophages (TAMs) in cancer patients is associated with worse OS (76). In comparison with normal macrophages, TAMs express high levels of HIF-2α, which seems to be an indicator of poor prognosis in cancer patients (77). Using *in vivo* models of acute inflammation, it has been shown that HIF-2α expression in macrophages is essential for inflammatory responses by regulating proinflammatory cytokine expression (78). Together, these studies show that HIF-2α tightly regulates macrophage functions, which in turn may impact the patient prognosis. We excluded studies in which only macrophage data were reported, since the main goal of this meta-analysis is to determine the prognostic role of HIF-2α expression in tumor cells. To note, 26% of the studies included in this meta-analysis stated HIF-2α reactivity in macrophages together with tumor cells.

The papers included in this meta-analysis were all published between 2001 and 2018, which is likely attributed to the fact that HIF-2α was first identified by independent groups a few years earlier (79, 80). First studies showed the importance of HIF-2α on the transcription of hypoxia-regulated genes such as VEGF in endothelial cells, fibroblasts, and epithelial cells. In addition, researchers described the novel role of HIF-2α in comparison with the already studied HIF-1α, its counteractive protein, in hypoxia and tissue homeostasis (80–83). The discovery of this new hypoxia-activated transcription factor encouraged research to further evaluate the role of HIF-2α *in vivo* and during development in mice. Their data show that HIF-2α displays a specific pattern of developmental expression at different embryonic stages (84). HIF-2α was thereby defined as a novel bHLH-PAS protein involved in the response to hypoxia showing overlapping but also independent roles with HIF-1α. Basic HLH (helix–loop–helix)–PER–ARNT–SIM (bHLH-PAS) proteins are a family of transcription factors, which respond to environmental signals such as low oxygen levels. This family of proteins is involved in dimerization, DNA binding, and signal transduction (85). Future studies demonstrated that HIF-2α was expressed in a much larger number of cell types (86). Apart from its regulatory role in normal tissue homeostasis, HIF-2α was seen to be commonly upregulated in a broad range of cancers and to contribute to multiple aspects of tumorigenesis such as altered metabolism, angiogenesis, epithelial–mesenchymal transition, and metastasis. This is supported by data showing that HIF-2α is involved in promoting resistance of tumor cells to several treatment modalities and increased patient mortality (87). The important role of HIF-2α in cancer prognosis is also supported by the results of this meta-analysis, which shows that patients with high HIF-2α expression have shorter OS. HIF-1α protein, in turn, has been shown to induce more aggressive phenotype in tumors cells by regulating similar cellular mechanisms (88). Therefore, assessment of oxygen-sensing proteins in tumors prior and/or during therapy may represent a powerful prognostic and predictive biomarker as well as important targets for new anticancer treatments, which warrants further investigations.

Importantly, there is also the risk of encountering publication bias since positive results are more likely to be published than negative ones. This meta-analysis identified a total of 49 studies of which 4 could not be included in final analysis because the HR could not be estimated due to incomplete reporting. Three of four studies stated non-significant association between HIF-2α and outcome (Table S1 in Supplementary Material). Including these four papers in the analysis might therefore decrease the magnitude of the prognostic value of HIF-2α expression reported here. Nevertheless, since the prognostic value of HIF-2α expression is highly statistically significant and the number of excluded studies is very low, we believe that the possible effect of publication bias on this association is negligible.

There are also other limitations in this meta-analysis. The approach of extrapolating the HRs could potentially introduce the source of bias. First, when it was not possible to extract HR directly from the article, survival curves were used to extract data to estimate HR following the method of Tierney et al. (70). Second, significant heterogeneity was found for most of the endpoints tested, which might confirm the high variability among studies. Reduced variability could be achieved by better stratifying tumors into high and low expressing. Third, the use of different antibodies with varying dilutions to detect HIF-2α, different staining protocols, different scoring methods, subcellular localization, and cutoff values may contribute to heterogeneity. Finally, due to the lack of papers referring to each specific endpoint, treatment modality, and/or organ site, it is difficult to set a robust outcome and achieve significant data for different subgroups. Therefore, more high-quality, large-sample, prospectively designed studies are needed to strengthen the prognostic and predictive relevance of HIF-2α in solid cancers.

The results presented here clearly indicate that HIF-2α expression is associated with worse prognosis in a global patient population and in some tumor sites. Altogether, the results of this meta-analysis support the development of a clinical test to determine patient prognosis and/or predict treatment outcome based on HIF-2α expression, although standardized protocols remains to be developed and validated. While potent inhibitors targeting HIF-2α are being translated into clinical trials for renal cancer (89) such predictive tests would be crucial in advancing anti-HIF2 inhibitors not only in renal cancer but also in other solid cancers.

## Author Contributions

Study was conceived and designed by ER, AY, and MV. Screening of papers and data extraction was performed by ER, AY, and AG. Statistical analyses were performed by RH. Writing of the first draft of the manuscript was performed by ER. AY, RH, AG, LD, and MV contributed to the writing of the manuscript.

## Conflict of Interest Statement

The authors declare that the research was conducted in the absence of any commercial or financial relationships that could be construed as a potential conflict of interest.
