# Supplementary material for: Prognostic Role of Hypoxia-Inducible Factor-2α Tumor Cell Expression in Cancer Patients: A Meta-Analysis
Source: Front Oncol. 2018 Jun 11;8:224. doi: 10.3389/fonc.2018.00224 (PMC6004384; doi:10.3389/fonc.2018.00224)
Supplement: Supplementary file 1 [file data_sheet_1.docx]

**Supplementary File 1.** Literature search strategy to identify papers for the meta-analysis that describe an association between tumoral HIF2α expression and prognosis.

**Research question:** What is the prognostic value of tumoral HIF2α expression in patients with solid tumors?

**Keywords identified as search terms:**

Prognosis, Cancer, and HIF2

**Search algorithm Pubmed:**

*Prognosis:*

(Prognos*) OR (prognostic value) OR (clinicopathological) OR (surviv*) OR (hazard) OR (disease-free) OR (“disease free”) OR (progression-free) OR (“progression free”) OR (Kaplan-meier) OR (“Kaplan meier”) OR (predict*) OR (outcome) OR (efficacy) OR (effective*)

*Cancer:*

(tumor) OR (tumors*) OR (tumor’s) OR (tumoral*) OR (“tumor associated”) OR (tumor-associated) OR (“tumor related”) OR (tumor-related) OR (tumorigen*) OR (tumorous*) OR (tumour*) OR (cancer) OR (cancers) OR (cancer’s) OR (cancerogen*) OR (cancera*) OR (cancer-associate*) OR (cancerigen*) OR (cancerno*) OR (cancero*) OR (cancerp*) OR (“cancer related”) OR (cancer-related) OR (*carcinoma) OR (*sarcoma) OR (neoplas*) OR (malignanc*) OR (melanoma)

*HIF2:*

(HIF2) OR (HIF-2) OR (EPAS1) OR (Endothelial PAS domain-containing protein 1) OR (Endothelial PAS domain protein 1) OR (HIF-2α) OR (HIF2α) OR (hypoxia-inducible factor 2α) OR (hypoxia-inducible factor-2α)

*Prognosis AND Cancer AND HIF2:*

((Prognos*) OR (prognostic value) OR (clinicopathological) OR (surviv*) OR (hazard) OR (disease-free) OR (“disease free”) OR (progression-free) OR (“progression free”) OR (Kaplan-meier) OR (“Kaplan meier”) OR (predict*) OR (outcome) OR (efficacy) OR (effective*)) AND ((tumor) OR (tumors*) OR (tumor’s) OR (tumoral*) OR (“tumor associated”) OR (tumor-associated) OR (“tumor related”) OR (tumor-related) OR (tumorigen*) OR (tumorous*) OR (tumour*) OR (cancer) OR (cancers) OR (cancer’s) OR (cancerogen*) OR (cancera*) OR (cancer-associate*) OR (cancerigen*) OR (cancerno*) OR (cancero*) OR (cancerp*) OR (“cancer related”) OR (cancer-related) OR (*carcinoma) OR (*sarcoma) OR (neoplas*) OR (malignanc*) OR (melanoma)) AND ((HIF2) OR (HIF-2) OR (EPAS1) OR (Endothelial PAS domain-containing protein 1) OR (Endothelial PAS domain protein 1) OR (HIF-2α) OR (HIF2α) OR (hypoxia-inducible factor 2α) OR (hypoxia-inducible factor-2α))

Hits: 626

**Search algorithm Embase:**

*Prognosis:*

(Prognos$) OR (prognostic value) OR (clinicopathological) OR (surviv$) OR (hazard) OR (disease-free) OR (“disease free”) OR (progression-free) OR (“progression free”) OR (Kaplan-meier) OR (“Kaplan meier”) OR (predict$) OR (outcome) OR (efficacy) OR (effective$)

*Cancer:*

(tumor) OR (tumors$) OR (tumor’s) OR (tumoral$) OR (“tumor associated”) OR (tumor-associated) OR (“tumor related”) OR (tumor-related) OR (tumorigen$) OR (tumorous$) OR (tumour$) OR (cancer) OR (cancers) OR (cancer’s) OR (cancerogen$) OR (cancera$) OR (cancer-associate$) OR (cancerigen$) OR (cancerno$) OR (cancero$) OR (cancerp$) OR (“cancer related”) OR (cancer-related) OR ($carcinoma) OR ($sarcoma) OR (neoplas$) OR (malignanc$) OR (melanoma)

*HIF2:*

(HIF2) OR (HIF-2) OR (EPAS1) OR (Endothelial PAS domain-containing protein 1) OR (Endothelial PAS domain protein 1) OR (HIF-2α) OR (HIF2α) OR (hypoxia-inducible factor 2α) OR (hypoxia-inducible factor-2α)

*Prognosis AND Cancer AND HIF2:*

((Prognos$) OR (prognostic value) OR (clinicopathological) OR (surviv$) OR (hazard) OR (disease-free) OR (“disease free”) OR (progression-free) OR (“progression free”) OR (Kaplan-meier) OR (“Kaplan meier”) OR (predict$) OR (outcome) OR (efficacy) OR (effective$)) AND ((tumor) OR (tumors$) OR (tumor’s) OR (tumoral$) OR (“tumor associated”) OR (tumor-associated) OR (“tumor related”) OR (tumor-related) OR (tumorigen$) OR (tumorous$) OR (tumour$) OR (cancer) OR (cancers) OR (cancer’s) OR (cancerogen$) OR (cancera$) OR (cancer-associate$) OR (cancerigen$) OR (cancerno$) OR (cancero$) OR (cancerp$) OR (“cancer related”) OR (cancer-related) OR ($carcinoma) OR ($sarcoma) OR (neoplas$) OR (malignanc$) OR (melanoma)) AND ((HIF2) OR (HIF-2) OR (EPAS1) OR (Endothelial PAS domain-containing protein 1) OR (Endothelial PAS domain protein 1) OR (HIF-2α) OR (HIF2α) OR (hypoxia-inducible factor 2α) OR (hypoxia-inducible factor-2α))

Hits: 10

Literature search was performed in Pubmed and Embase on the 1th of February 2018.
