# Supplementary material for: Prognostic Role of Hypoxia-Inducible Factor-2α Tumor Cell Expression in Cancer Patients: A Meta-Analysis
Source: Front Oncol. 2018 Jun 11;8:224. doi: 10.3389/fonc.2018.00224 (PMC6004384; doi:10.3389/fonc.2018.00224)
Supplement: Supplementary file 2 [file data_sheet_2.docx]

**Supplementary File 2.** Adjusted version of the Newcastle-Ottawa Scale to assess the quality of the included papers. In brief, for each criteria a single option can be registered. The amount of answers with stars behind them are counted and the total number of stars is a measurement of the study quality.

**NEWCASTLE – OTTAWA QUALITY ASSESSMENT SCALE**

**Adjusted version**

Note: A study can be awarded a maximum of one star for each numbered. When criteria are not reported no star can be awarded for that category.

**First author:** .......................................................................**Year of publication:** ………………

**Selection**

1) Representativeness of the cohort

a. Truly representative of the average patient population ★

b. Somewhat representative of the average patient population ★

c. Selected group of patients based on certain criteria

d. No description of the derivation of the cohort

**Grouping variable**

1) Was the measurement performed blindly from the outcome

a. Yes ★

b. No

c. Not stated

2) How many persons performed the scoring

a. More than one ★

b. One

c. Not stated

3) Was scoring between investigators performed independently

a. Yes ★

b. No

c. Not stated

**Outcome**

1) Assessment of outcome

a. Independent blind assessment ★

b. Record linkage ★

c. Self-report

d. No description

2) Was follow-up long enough for outcomes to occur

a. Yes ★

b. No

3) Adequacy of follow-up of cohorts

a. Complete follow-up – all subjects accounted for ★

b. Subjects lost to follow-up unlikely to introduce bias ★

(Equally distributed/small numbers)

c. Follow-up rate unevenly distributed or large numbers lost

d. No statement
