# Supplementary material for: Prognostic Role of Hypoxia-Inducible Factor-2α Tumor Cell Expression in Cancer Patients: A Meta-Analysis
Source: Front Oncol. 2018 Jun 11;8:224. doi: 10.3389/fonc.2018.00224 (PMC6004384; doi:10.3389/fonc.2018.00224)
Supplement: Supplementary file 3 [file data_sheet_3.PDF]

Supplementary File 1. Literature search strategy to identify papers for the meta-analysis that describe an association between tumoral HIF2α expression and prognosis.

Research question: What is the prognostic value of tumoral HIF2α expression in patients with solid tumors?

Keywords identified as search terms:

Prognosis, Cancer, and HIF2

Search algorithm Pubmed:

Prognosis:

(Prognos*) OR (prognostic value) OR (clinicopathological) OR (surviv*) OR (hazard) OR (disease-free) OR ("disease free") OR (progression-free) OR ("progression free") OR (Kaplan-meier) OR ("Kaplan meier") OR (predict*) OR (outcome) OR (efficacy) OR (effective*)

Cancer:

(tumor) OR (tumors*) OR (tumor's) OR (tumoral*) OR ("tumor associated") OR (tumor-associated) OR ("tumor related") OR (tumor-related) OR (tumorigen*) OR (tumorous*) OR (tumour*) OR (cancer) OR (cancers) OR (cancer's) OR (cancerogen*) OR (cancera*) OR (cancer-associate*) OR (cancerigen*) OR (cancerno*) OR (cancero*) OR (cancerp*) OR ("cancer related") OR (cancer-related) OR (*carcinoma) OR (*sarcoma) OR (neoplas*) OR (malignanc*) OR (melanoma)

HIF2:

(HIF2) OR (HIF-2) OR (EPAS1) OR (Endothelial PAS domain-containing protein 1) OR (Endothelial PAS domain protein 1) OR (HIF-2α) OR (HIF2α) OR (hypoxia-inducible factor 2α) OR (hypoxia-inducible factor-2α)

Prognosis AND Cancer AND HIF2:

((Prognos*) OR (prognostic value) OR (clinicopathological) OR (surviv*) OR (hazard) OR (disease-free) OR ("disease free") OR (progression-free) OR ("progression free") OR (Kaplan-meier) OR ("Kaplan meier") OR (predict*) OR (outcome) OR (efficacy) OR (effective*)) AND ((tumor) OR (tumors*) OR (tumor's) OR (tumoral*) OR ("tumor associated") OR (tumor-associated) OR ("tumor related") OR (tumor-related) OR (tumorigen*) OR (tumorous*) OR (tumour*) OR (cancer) OR (cancers) OR (cancer's) OR (cancerogen*) OR (cancera*) OR (cancer-associate*) OR (cancerigen*) OR (cancerno*) OR (cancero*) OR (cancerp*) OR ("cancer related") OR (cancer-related) OR (*carcinoma) OR (*sarcoma) OR (neoplas*) OR (malignanc*) OR (melanoma)) AND ((HIF2) OR (HIF-2) OR (EPAS1) OR (Endothelial PAS domain-containing protein 1) OR (Endothelial PAS domain protein 1) OR (HIF-2α) OR (HIF2α) OR (hypoxia-inducible factor 2α) OR (hypoxia-inducible factor-2α))

Hits: 626

Search algorithm Embase:

Prognosis:

(Prognos$) OR (prognostic value) OR (clinicopathological) OR (surviv$) OR (hazard) OR (disease-free) OR ("disease free") OR (progression-free) OR ("progression free") OR (Kaplan-meier) OR ("Kaplan meier") OR (predict$) OR (outcome) OR (efficacy) OR (effective$)

Cancer:

(tumor) OR (tumors$) OR (tumor's) OR (tumoral$) OR ("tumor associated") OR (tumor-associated) OR ("tumor related") OR (tumor-related) OR (tumorigen$) OR (tumorous$) OR (tumour$) OR (cancer) OR (cancers) OR (cancer's) OR (cancerogen$) OR (cancera$) OR (cancer-associate$) OR (cancerigen$) OR (cancerno$) OR (cancero$) OR (cancerp$) OR ("cancer related") OR (cancer-related) OR ($carcinoma) OR ($sarcoma) OR (neoplas$) OR (malignanc$) OR (melanoma)

HIF2:

(HIF2) OR (HIF-2) OR (EPAS1) OR (Endothelial PAS domain-containing protein 1) OR (Endothelial PAS domain protein 1) OR (HIF-2α) OR (HIF2α) OR (hypoxia-inducible factor 2α) OR (hypoxia-inducible factor-2α)

Prognosis AND Cancer AND HIF2:

((Prognos$) OR (prognostic value) OR (clinicopathological) OR (surviv$) OR (hazard) OR (disease-free) OR ("disease free") OR (progression-free) OR ("progression free") OR (Kaplan-meier) OR ("Kaplan meier") OR (predict$) OR (outcome) OR (efficacy) OR (effective$)) AND ((tumor) OR (tumors$) OR (tumor's) OR (tumoral$) OR ("tumor associated") OR (tumor-associated) OR ("tumor related") OR (tumor-related) OR (tumorigen$) OR (tumorous$) OR (tumour$) OR (cancer) OR (cancers) OR (cancer's) OR (cancerogen$) OR (cancera$) OR (cancer-associate$) OR (cancerigen$) OR (cancerno$) OR (cancero$) OR (cancerp$) OR ("cancer related") OR (cancer-related) OR ($carcinoma) OR ($sarcoma) OR (neoplas$) OR (malignanc$) OR (melanoma)) AND ((HIF2) OR (HIF-2) OR (EPAS1) OR (Endothelial PAS domain-containing protein 1) OR (Endothelial PAS domain protein 1) OR (HIF-2α) OR (HIF2α) OR (hypoxia-inducible factor 2α) OR (hypoxia-inducible factor-2α))

Hits: 10

Literature search was performed in Pubmed and Embase on the 1th of February 2018.

Supplementary File 2. Adjusted version of the Newcastle-Ottawa Scale to assess the quality of the included papers. In brief, for each criteria a single option can be registered. The amount of answers with stars behind them are counted and the total number of stars is a measurement of the study quality.

NEWCASTLE – OTTAWA QUALITY ASSESSMENT SCALE
Adjusted version

Note: A study can be awarded a maximum of one star for each numbered. When criteria are not reported no star can be awarded for that category.

First author: ....**Year of publication:** ………………

Selection

1) Representativeness of the cohort
    a. Truly representative of the average patient population ★
    b. Somewhat representative of the average patient population ★
    c. Selected group of patients based on certain criteria
    d. No description of the derivation of the cohort

Grouping variable

1) Was the measurement performed blindly from the outcome
    a. Yes ★
    b. No
    c. Not stated
2) How many persons performed the scoring
    a. More than one ★
    b. One
    c. Not stated
3) Was scoring between investigators performed independently
    a. Yes ★
    b. No
    c. Not stated

Outcome

1) Assessment of outcome
    a. Independent blind assessment ★
    b. Record linkage ★
    c. Self-report
    d. No description
2) Was follow-up long enough for outcomes to occur
    a. Yes ★
    b. No

3) Adequacy of follow-up of cohorts

   a. Complete follow-up – all subjects accounted for ★
   b. Subjects lost to follow-up unlikely to introduce bias ★
      (Equally distributed/small numbers)
   c. Follow-up rate unevenly distributed or large numbers lost
   d. No statement

Supplementary Table 1.

Study	**Endpoint**	**Organ**	**Patients**	**Comments**
**Nanni 2009**	DSS	Prostate	88	''While no correlation was found between HIF-2α expression and DSS''.
**Liang 2011**	DSS	Head and Neck	89	''However, HIF- 2a had no significant association with either overall survival (P = 0.195, Fig. 1b) or disease-free survival (P = 0.356).'' Only OS data shown.
**Wang 2015**	OS	Pancreas	90	Only P value reported (0.0078).
**Hui 2002**	OS	Head and Neck	89	''No significant association between HIF-2, CA IX, or VEGF expression with respect to OS or PFS was found".
**Hui 2002**	PFS	Head and Neck	89	''No significant association between HIF-2, CA IX, or VEGF expression with respect to OS or PFS was found".

Supplementary Table 2

**Study**	**Subcellular localization**	**HR(95%CI)**
Biswas 2012	Nuclear	0.77 (0.44 – 1.33)
Kroeger 2014	Nuclear	**0.61 (0.39 – 0.97)**
Kroeger 2014	Cytoplasm	**2.33 (1.6 – 3.39)**
Liu 2018	Cytoplasm	2.27 (0.84 – 6.17)

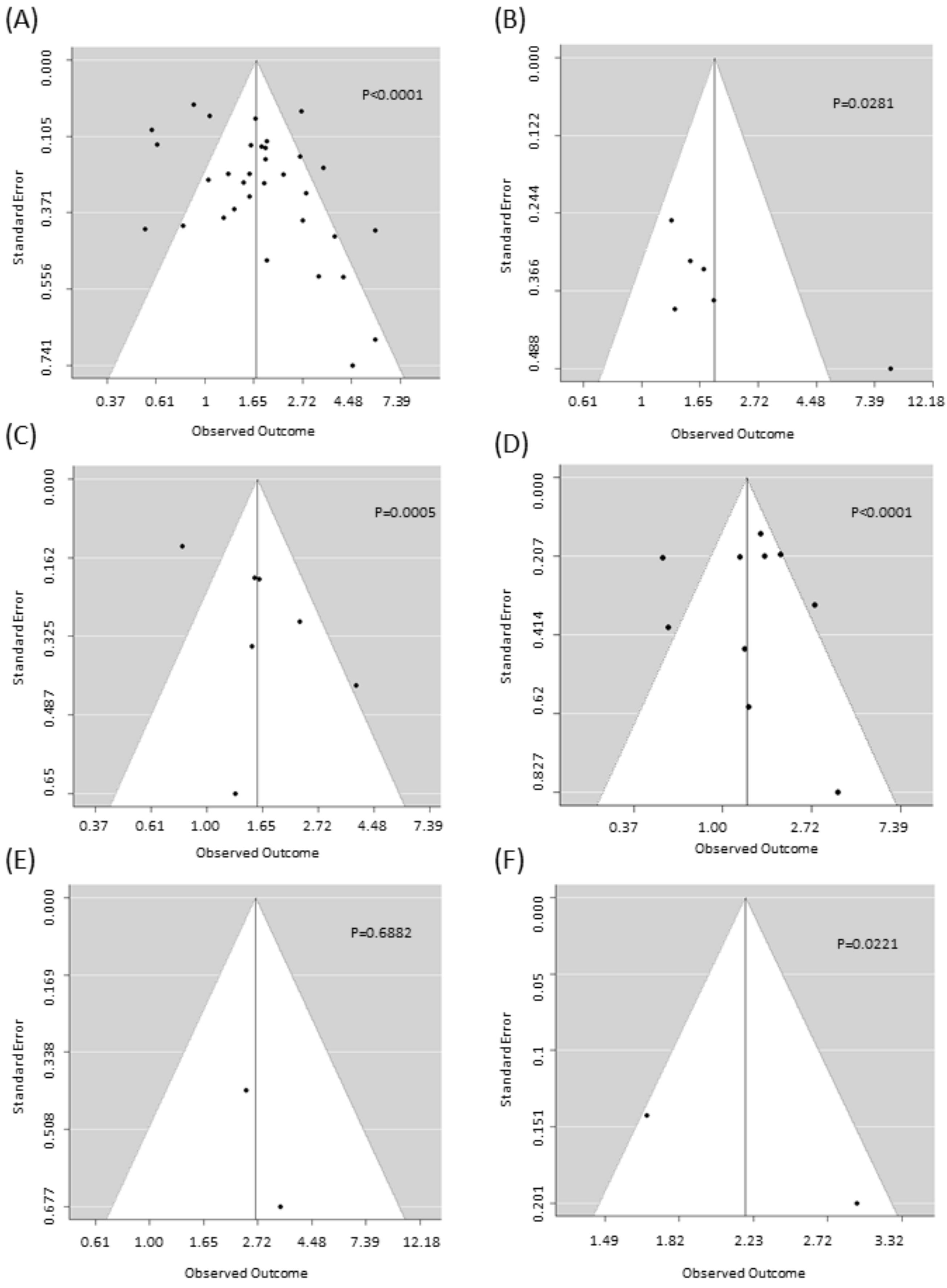

Supplementary Figure 1 Funnel plots of papers reporting on OS (A), DFS (B), DSS (C), LC (D), MFS (E), and PFS (F). The x-axis represents the HR for each paper with the corresponding standard error on the y-axis. Heterogeneity is significant for OS (A), DFS (B), LC (C), DSS (D), and PFS (F).
